# Assessing Factors That Influence Healthcare Provider Attitudes and Practices regarding Place-Based Exercise Prescriptions: Results of Principal Components Analysis of a Newly Developed Survey Instrument

**DOI:** 10.1155/2020/5084053

**Published:** 2020-05-11

**Authors:** Carissa Smock, Sheryl L. Chatfield

**Affiliations:** ^1^Health Services North central University, 11335 Torrey Pines Road La Jolla, CA 92037, USA; ^2^College of Public Health, Kent State University, Moulton Hall, 800 Hilltop Drive, Kent, OH 44242, USA

## Abstract

**Introduction:**

The purpose of this paper is to describe development and preliminary assessment of an instrument designed to assess facilitators and barriers of provider-provided, place-based exercise prescriptions, including provider attributes, perceptions, knowledge, and resource needs. Although the American Medical Association-Supported “Exercise is Medicine” initiative encourages the practice of exercise prescription among member providers, only a small proportion engages in this practice. Additionally, little is known about the role of place-based exercise prescriptions, although access to physical activity resources differs based on residence, access to transportation, income, and other factors. To utilize potential for prescriptions to encourage physical activity, better understanding of the role of place is essential.

**Methods:**

Previously validated and newly developed items were combined to create an 88-item survey that was administered to 166 healthcare providers.

**Results:**

Results of principal components analysis suggested a five-factor structure; three factors—provider belief in exercise benefits, provider training needs, and place-based concerns—demonstrated high internal consistency. Factors demonstrating low internal consistency included provider attitudes about their role in exercise prescription and providers' perceptions of patient barriers.

**Conclusions:**

Following this stage in survey validation, the 88-item developed survey could be shortened by eliminating items with low loadings. Providers may be more receptive to a shorter instrument, which could facilitate reliability and validity testing of a revised instrument. Further steps to validate the instrument include assessing consistent responses over time and considering predictive ability of the survey as an additional measure of validity. Results from the initial survey administration indicate that providers' lack of training regarding how to prescribe exercise and lack of knowledge of safe, affordable, or proximate locations for patients to engage in prescribed exercise present barriers to wider use of exercise prescriptions. Community-clinical linkages which network providers with area physical activity and exercise resources may present a partial solution. Knowledge of safe, affordable, or proximate locations for patients to engage in prescribed exercise presents a barrier to place-based exercise prescriptions.

## 1. Introduction

Multiple researchers have reported that physical inactivity in the US is associated with increased healthcare costs. Carson et al. [1] integrated data regarding health status and healthcare expenses for 51,165 adult Americans and concluded 12.5% of direct healthcare expenses are associated with insufficient participation in physical activity. Additionally, within this substantial sample, 54.4% of respondents reported being inactive or insufficiently active. One approach that has a potential of encouraging participation in physical activity and exercise is the use of healthcare provider-initiated exercise referrals and exercise prescriptions. Exercise prescriptions (EPs) combine aspects of government and association-provided recommendations with exercise counseling to formulate a patient-specific protocol. Research evidence suggests that EPs and other community health counseling efforts reflect cost-effective methods to increase individuals' regular participation in exercise [2].

Systematic exercise recommendation and counseling efforts in the US began during the 1970s; the American College of Sports Medicine [3] provided the first population-based, dose-specific exercise recommendations in 1978. These included that all adults should engage in exercise for at minimum 3–5 days per week for a duration of 15 to 60 minutes per day and should perform exercise at a target heart rate between 50 and 85% of their maximum predicted heart rate. The US Preventive Services Task Force (USPSTF) developed the first national guidelines for exercise counseling in 1989 [4]. These guidelines encouraged physicians to incorporate exercise participation questions as part of patient history information gathering, to use this information to identify patients who did not meet the ACSM guideline, and to educate patients about disease prevention potential of exercise. Physicians were additionally encouraged by USPSFT to guide patients in choosing appropriate exercise type, intensity, duration, and frequency, to monitor patient compliance to exercise participation, and to work with individual patients to identify barriers and encourage facilitators such as provision of support from significant others.

The specific physical activity guidelines for Americans were revised in 1995 to recommend 30 minutes or more of moderate-intensity physical activity on most days of the week [5]. In 2007, ACSM and the American Heart Association (AHA) further revised and refined recommendations to specifically address frequency, intensity, and duration of aerobic exercise and to recommend addition of strength training exercise [6]. Also in 2007, the American Medical Association (AMA) and ACSM colaunched the Exercise is Medicine (EIM) initiative, which was endorsed by the American Academy of Family Physicians [7]. Healthcare provider exercise prescription tools available on the EIM website include the Healthcare Providers' Action Guide, an exercise prescription protocol that includes referral to an ACSM-certified exercise specialist and exercise clearance and exercise readiness screening forms. In 2010, the US National Physical Activity Plan (NPAP) was introduced by a coalition of public and private sector organizations, including ACSM, AHA, AMA, and others, to develop a consensus plan for exercise recommendations and promotion [8]. The uniting goal of EIM and NPAP is to make exercise a vital sign, analogous to heart rate, body temperature, and blood pressure, that is monitored by healthcare providers with the same level of priority as the more traditional vital signs. However, despite these many efforts to encourage and educate primary care providers to prescribe exercise, only an estimated 14% of primary care providers report that they prescribe exercise to roughly 50% of their patients [9].

Clearly, recommendations for exercise and associated EP resources have become increasingly explicit as regards type, duration, intensity, and frequency of exercise. However, guidance regarding exercise access points, such as public, private, indoor, or outdoor facilities and resources, has not been consistently incorporated into EP practices or guidelines, although prior researchers (e.g., [10, 11]) have demonstrated that place is a factor that plays an influential role as a barrier or a facilitator of exercise. According to [12], stronger links between the healthcare system and readily available exercise access points, including not only hospital owned fitness facilities, but also community centers, parks, and trails, might help the medical community promote activities that have a higher likelihood of becoming a permanent part of a given patient's life. Among the barriers reported by providers that prevent their frequent and enthusiastic participation in provision of EPs is a sense of doubt that patients will comply [13]. This emphasizes the importance of use of processes, including place-based EPs that have most potential to encourage patient compliance, which will increase provider confidence in EPs and their usage.

Although authors have explored the general attitudes, facilitators, and barriers of providers' general EP practices (e.g., [14, 15]), to date, no authors have investigated factors, including physician attributes, that influence physician attitudes and practices related to place-based EPs. The purpose of this paper is to describe initial development and results from exploratory factor analysis of an assessment, aimed at healthcare providers, that explores physicians' perceptions, barriers, knowledge, and personal practices considered in the context of place-based EPs. Because some physicians have previously reported lack of exercise expertise as a barrier toward the practice of exercise prescription [16], additional goals of this instrument included assessment of provider attitudes toward referrals to exercise professionals and identification of other provider-recommended resources to facilitate their use of EPs.

## 2. Materials and Methods

### 2.1. Instrument Development

Based on review of prior literature consistent with the aims of this study, the following three categories were identified to form the basis for the assessment instrument. (1) What are the general provider attitudes, knowledge, confidence, perceived barriers, and practices related to providing exercise referrals and prescriptions to their patients? (2) What are the characteristics of providers who currently refer patients to a professional or place for exercise? (3) What do providers believe will increase their likelihood of referring patients to a professional or a place for exercise?

In order to identify or develop specific items, these broad categories were further divided into eight subcategories, including (1) provider attitudes about general benefits of exercise, (2) provider beliefs about their role as exercise advocates for patients, (3) provider identified perceived barriers to EP, (4) provider knowledge related to exercise recommendations, (5) current provider EP practices, including use of and attitudes toward place-based EPs, (6) provider continuing education needs related to EPs, and (7) providers' own exercise habits, and (8) demographic information.

Categories 1–3 address constructs; category 4 assessed current knowledge; categories 5, 7, and 8 reflect provider practices and attributes. Provider exercise habits have been shown to have a positive influence on exercise counseling and provision of EPs [17], comprising one of the attribute categories. Because provider lack of knowledge has been offered as a barrier to provision of EPs, category 6 items provided an opportunity to solicit information from healthcare providers regarding their interest in continuing education.

Wherever possible, items and scales from existing surveys with established psychometric properties were used. The survey is included in the Appendix; existing items and sources are shown in Table 1. When no preexisting question or scale could be identified to assess a construct or attribute, new items were developed based on review of relevant literature.

The pilot instrument developed through this process was administered to a healthcare provider, a medical student, a doctoral student in a college of public health, and a faculty member in a college of public health. The goals of pilot administration were to assess clarity of items and to derive a time estimate for survey completion. Based on comments from the pilot group, debriefing meetings were held with the provider and the faculty member to further refine instrument language. Time for completion averaged 10 minutes and the pilot participants agreed this was an appropriate estimate to share with participants. This also aligned with literature exploring physician survey completion rates [26]. The refined version of the survey, included in the Appendix in Supplementary Materials consisted of 88 items.

### 2.2. Survey Administration

The pilot study population comprised currently practicing healthcare providers who interact with individual patients who have potential to provide exercise counseling or write EPs. As this was a newly developed instrument, there were no available prior data on which to base power analysis or effect and sample size calculations. Additionally, the primary goal of this specific study was to develop and assess an instrument rather than to offer assertions regarding generalizability of the findings. Therefore, our priority aim in recruitment was to solicit completion of as many surveys as possible from individuals who represented the population of interest.

Participants were recruited from two local hospitals (*n* = 223) and from an email list of university alumni of a nurse provider degree program (*n* = 1545). A mixed-mode distribution method was used, with initial distribution of questionnaires and informed consent distributed via email and mail. One week after initial distribution, a follow-up paper version of the survey was sent. Two weeks later, any remaining providers who had not yet responded were sent an additional email request with a link to the survey. No personally identifying information was requested from participants; although participants were offered incentives in the form of 10 US dollars coffee gift card, information on receiving the incentive was gathered via a web link not associated with the survey process. A university Institutional Review Board approved the survey and recruitment process.

### 2.3. Data Analysis

Principal components analysis (PCA) was completed on the survey items related to provider attitudes about benefits of exercise, beliefs about their role as exercise advocates, barriers to exercise counseling/EPs, knowledge about exercise recommendations, and current practices with respect to exercise prescriptions. The goals of PCA were twofold and follow the practical uses of PCA as described by [27] including that this is an exploratory study trying to maximize the variance (unique, shared, and error variance) as opposed to only shared variance in factor analysis. The first goal was to identify independent sets of correlated items that comprised categories or factors. Items were grouped in the survey by construct and the PCA process revealed the extent to which these groupings are appropriate by identifying the bivariate correlations among grouped items and correlations between items that were not grouped together. This determined whether or not these factors correspond to the underling theoretical constructs we attempted to measure using this survey instrument. The results of factor extraction followed the norm as each extracted factor had an eigenvalue greater than 1.0. The second goal of the PCA process was to facilitate reduction of survey items by identifying and retaining the best measures of each construct.

Identification of principal components was conducted by examining the resulting scree plot, eigenvalues, and variance. Direct oblimin (oblique) rotation was used as each factor had simple structure: few with high loadings and the remainder being zero or close to zero [28]. For adequate convergent validity, it is expected that items belonging to a common construct should exhibit factor loadings of 0.60 or higher on a single factor. Cronbach's alpha was used to assess consistency among identified high loading indicators for each component. As it should be noted that alpha does not include evidence of dimensionality, therefore it cannot be determined if this instruments' subscales are supported by the factor analysis.

## 3. Results

Substantially completed surveys were received from 166 provider participants, or 74% of those contacted. Response rate from the nurse provider alumni was disappointingly low (*n* = 8) and was attributed to postgraduation disuse of official university email addresses, the method used to distribute the surveys.

Five underlying constructs were identified, each containing at least three items; three items are the minimum number of indicators required per construct [27]. Following a scale reliability analysis determined that the identified scales were sufficiently reliable to justify using them in the analysis. Based on Cronbach's alpha, two out of the five scales had low internal consistency based on .80 as a cutoff.  Table 2 shows components with the three highest loading indicator items and associated loadings.  Table 3 shows Cronbach's alpha, the reliability statistic, for each component. The provider belief of exercise benefits (Factor 2), location/place-based (Factor 3), and training and guidelines (Factor 1) scales resulted in Cronbach's alphas of >0.80. The providers' attitudes about their role in exercise practices (Factor 5) and patient factor barriers (Factor 4) resulted in Cronbach's alphas <0.80. Additional details about data analysis and access to deidentified data are available by contacting the first author.

## 4. Discussion

The purpose of this research was to assess a newly developed 88-item questionnaire designed to assess healthcare provider attitudes toward exercise prescriptions. Although the instrument was designed to address eight categories, analysis of the first administration suggested an underlying 5-component structure. Each component of the developed instrument revealed consistencies and inconsistencies that begin to better describe provider attitudes, knowledge, confidence, perceived barriers, and practices toward place-based EPs.

The items with higher internal consistency, including provider need for training, provider belief of benefits, and provider appreciation of the role of place, taken as a whole, suggest that providers are invested in the idea of exercise and appreciative of the benefits, but may lack confidence in the adequacy of their preparation and training to appropriately address this issue with patients. That said, some research suggests that patients are up to five times more likely to increase exercise when prescribed by a provider [29]. Primary care providers also have contact with the broadest range of repeated patient visits over time and can engage in both primary and secondary prevention through exercise prescriptions [16]. This suggests that there is tremendous potential for providers to impact health-promoting exercise participation among their patients.

Other providers, such as specialists, may only see patients for acute care; however, these providers are still viewed as an authority by patients and, therefore, influential in exercise adoption. These specialists may better understand the physical limitations of patients with specific disease states and risk factors [3]. Additionally, patients are increasingly apt to increase exercise when a provider indicates that they are at risk for a disease, as opposed to concerns based solely on weight [30]. However, the inconsistency in the types of patients providers refer suggests that providers should be further engaged in primary prevention so that patients are receiving exercise recommendations before they develop chronic conditions such as obesity and diabetes, rather than after.

To partially address providers' lack of knowledge or confidence in administering exercise prescriptions, it is important to consider that providers may be overlooking their opportunity to refer patients to other resources, including park and recreation facilities and exercise professionals. These referrals, when offered in association with a general recommendation to increase participation in physical activity, may serve to better encourage patient uptake. Admittedly, one challenge in connecting patients to physical activity access points within their community is finding links that provider can depend on to (1) schedule with the patient, (2) follow up with the patient, and (3) report uptake of the recommended community resource so it can be recorded in the patient's record. “Stop smoking” programs have seen increases in quitline use from just 2–3% to as much as 50% by including a follow-up phone call [31]. Potential for community physical activity experts from locations such as parks, YMCA's, and bike programs can provide not only valuable clinical use for health outcomes, but also valuable research information to determine effective interventions within the clinical world for physical activity behaviour change. Therefore, it is important to better understand factors that influence employees at parks, YMCA's, and bike programs attitudes and practices regarding place-based exercise prescriptions as well. Adaption of this instrument could be used in future research to measure these factors. Because physical activity referrals and prescriptions do not occur frequently due to provider, time, lack of training, and lack of resources to refer the patient, community resources have the potential to link the patient to the desired clinical outcomes and provide the scheduling and follow-up needed for effective uptake.

## 5. Conclusion

This study provides description of initial development and assessment of a survey, as part of the process to refine a survey suitable for future use in larger samples of healthcare providers. Administration of this survey with this sample suggested five underlying factors that might be represented by three survey items. The next step in the process of establishing psychometric properties for this survey is administration of the abbreviated survey to an adequate sample to ensure the factor structure and reliability coefficient indicators continue to be acceptable [32]. This step will need to be repeated if use of the survey in different contexts is desired. Reliability may be improved by additional administration over time (i.e., test-retest reliability). Following revisions indicated by this readministration, additional validity tests described by Boateng et al. include assessment of predictive validity, via regression models to assess strength of association(s) among variables and outcomes and construct validity. The latter can be helpfully assessed by comparing alternative measures and alternative ways of measuring the identified factors [33], such as gathering qualitative interview or observational data during implementation of an intervention planned to encourage provider use of place-based EPs.

Based on assessment of provider attitudes, knowledge, confidence, perceived barriers, and practices related to providing exercise referrals and prescriptions to their patients, it appears providers are in need of increased knowledge to increase confidence and decrease barriers. Despite the known health risks of physical inactivity, prescribing exercise to patients is not a common practice among nurse providers and primary care providers in this sample. Results of PCA suggest that the assessed 88-item instrument might be reduced so that fewer items yield adequate results. Administration of a shorter survey may improve response rates and contribute to improved understanding of provider perceptions in various contexts. Additionally, adaption of this instrument could be used in future research to better understand factors that influence employees at parks, YMCA's, and bike programs attitudes and practices regarding placed-based exercise prescriptions to increase feasibility through community partnerships.

## Figures and Tables

**Table 1:** Sources for survey items.

Category	Source(s)	Survey question(s)
Clinician belief of exercise benefits	[13, 18]	1–4
Clinician attitude about their role in exercise practices	[13, 19, 20]	5–12
Clinician barriers	[12, 19–22]	48–65
Knowledge of existing exercise recommendations	[20]	13–17
Current EP practices	[3, 16, 19, 20, 23]	18–47
Needs for EP continuing education	[3, 13, 24]	66–70
Clinician exercise habits	[25]	71–77

**Table 2:** Components and loadings.

Survey item	Component				
	1	2	3	4	5
Q4.	0.009	0.307	−0.081	0.103	**0.596**
Q3.	0.028	**0.888**	−0.064	0.077	0.001
Q2.	0.072	**0.867**	−0.025	0.076	0.015
Q1.	−0.062	**0.839**	−0.040	0.058	0.072
Q50.	0.014	−0.010	0.113	**0.755**	0.068
Q52.	0.150	−0.030	0.077	**0.695**	0.135
Q 53.	**0.713**	−0.016	0.176	0.090	0.245
Q56.	**0.707**	−0.034	0.386	0.028	0.127
Q 57	**0.707**	0.007	0.432	0.038	0.224
Q 60.	0.228	−0.126	**0.745**	0.011	0.140
Q 61.	0.282	−0.036	**0.778**	0.094	0.006
Q62.	0.230	−0.017	**0.797**	0.063	0.104
Q64.	−0.088	0.084	0.358	**0.559**	−0.084
Q7.	−0.063	−0.058	0.079	0.104	**0.800**
Q8	0.151	0.221	0.229	−0.101	**0.694**

Extraction method: principal component analysis. Rotation method: varimax with Kaiser normalization.

**Table 3:** Factors and reliability statistics.

Factor	Summary	Cronbach's alpha
1	Provider training needs	0.843
2	Benefits of exercise	0.877
3	Place-based exercise	0.866
4	Patient barriers	0.584
5	Provider role	0.625

## Data Availability

Access to individual participant responses to surveys used to support the findings of this study is not available based on limitations of comply with informed consent provided by participants as required by the Kent State University Institutional Review Board. Deidentified aggregate data are available from Dr. Carissa Smock, csmock@ncu.edu, upon request.

## Supplementary Materials

Supplementary Materials This section includes the Appendix with questionnaire.
