# Supplementary material for: Assessing Factors That Influence Healthcare Provider Attitudes and Practices regarding Place-Based Exercise Prescriptions: Results of Principal Components Analysis of a Newly Developed Survey Instrument
Source: Adv Prev Med. 2020 May 11;2020:5084053. doi: 10.1155/2020/5084053 (PMC7238351; doi:10.1155/2020/5084053)
Supplement: Supplementary Materials — This section includes the Appendix with questionnaire. [file 5084053.f1.pdf]

APPENDIX

Questionnaire

Please answer the following items with respect to your practice. Please circle your response(s) for each item:

As you answer, please keep in mind that exercise prescriptions are defined, like all prescriptions are, as having a type and dose, dosing frequency, a duration of treatment as a therapeutic goal, anticipated adverse effect, and written as a script.

Exercise counseling is defined as asking and informally and orally recommending exercise that may or may not include type and dose.

	Please state the level to which you agree or disagree with the following statements (PLEASE CIRCLE ONE for each statement):	**Disagree Strongly**	**Disagree Slightly**	**Neutral**	**Agree Slightly**	**Agree Strongly**
**1**	Exercise is beneficial in preventing chronic diseases.	1	2	3	4	5
**2**	Exercise can yield benefits for individuals of any age.	1	2	3	4	5
**3**	Exercise is beneficial in improving mental health.	1	2	3	4	5
**4**	Exercise has additional benefits if preformed outdoors.	1	2	3	4	5
	**<u>Provider role:</u>**					
**5**	Ascertaining information on preventive health behaviors, including exercise, is an important part of patient history-taking.	1	2	3	4	5
**6**	Practitioners should be proactive in prescribing exercise for all patients.	1	2	3	4	5

**7**	There should be required education and certification for providers to deliver exercise prescriptions.	1	2	3	4	5
**8**	Providers should advocate for policies that support exercise prescriptions.	1	2	3	4	5
**9**	Exercise <u>counseling</u> is stressed in your practice.	1	2	3	4	5
**10**	Exercise <u>prescription</u> is stressed in your practice.	1	2	3	4	5
**11**	What percentage of patients do you believe would increase their exercise levels if you were to counsel about or prescribe exercise?	1<br>1-<br>20%	2<br>21-<br>40%	3<br>41-<br>60%	4<br>61-<br>80%	5<br>81-<br>100%
**12**	On a scale of 1-10, how likely are you to advocate for exercise Clinical Practice Guidelines? (Circle ONE response)	1 2	3 4	5 6	7 8	9 10

<u>Exercise Perspectives:</u>

		--------Minutes-------			
**13**	The US Physical Activity Guidelines recommend that adults get how many minutes of moderate physical activity per week? (Circle ONE response)	60	90	120	300
**14**	The US Physical Activity Guidelines recommend that adults engage in bone and muscle strengthening activities at least how many times per week? (Circle ONE response))	1	2	3	4+

15 Please CHECK ALL exercise initiatives you are aware of

☐ Personal Health Investment Today Act
☐ Workplace Health Improvement Program Act
☐ Physical Inactivity as a Disease
☐ Place as a Vital Sign
☐ Exercise as a Vital Sign
☐ Moving Outdoor in Nature Act
☐ Exercise is Medicine
☐ Other________________________________

**16**	On a scale of 1-10, ('1' being not knowledgeable and '10' being extremely knowledgeable) how would you rate your knowledge in regard to exercise prescriptions?	1	2	3	4	5	6	7	8	9	10
**17**	On a scale of 1-10, ('1' being not confident and '10' being extremely confident) how would you rate your confidence in regards to writing exercise prescriptions?	1	2	3	4	5	6	7	8	9	10

<u>Exercise prescription practices:</u>

**18**	Do you ask/inquire about the exercise behaviors of your patients?<br>**If no, skip to #22 (next page)**	Yes	No

**19**	If yes, with which percent of patients do you inquire? (Circle ONE response)	1<br>1-<br>20%	2<br>21-<br>40%	3<br>4-<br>60%	4<br>61-<br>80%	5<br>81-<br>100%

20 If yes, with what target group(s) of patients do you ask/inquire about physical activity/exercise?
(CHECK ALL THAT APPLY)

- ☐ All patients
- ☐ At risk for diabetes
- ☐ At risk for heart disease
- ☐ At risk for high blood pressure
- ☐ At risk for cancer
- ☐ At risk for mental illness
- ☐ Diagnosed with diabetes
- ☐ Diagnosed with heart disease
- ☐ Diagnosed with high blood pressure
- ☐ Diagnosed with cancer
- ☐ Diagnosed with mental illness
- ☐ Other ___________________

21 If yes, what motivates you to ask/inquire about exercise? (CHECK ALL THAT APPLY)

- ☐ Importance to health
- ☐ Quality Incentives
- ☐ Other
- ☐ A program that you refer patients to called __________

**22**	Do you use electronic medical records (EMR) in your practice? (CIRCLE ONE)<br>**If no, skip to #24**	Yes	No
**23**	Does your EMR include a prompt to document exercise levels? (CIRCLE ONE)	Yes	No
**24**	Do you document exercise of your patients? (CIRCLE ONE)<br>**If no, skip to #28**	Yes	No

		1	2	3	4	5
**25**	If yes, what percentage of patients have you documented information on exercise? (CIRCLE ONE)	1-20%	21-40%	41-60%	61-80%	81-100%

26 If yes, with which target group(s) of patients do you record exercise? (CHECK ALL THAT APPLY)

☐ All patients
☐ At risk for diabetes
☐ At risk for heart disease
☐ At risk for high blood pressure
☐ At risk for cancer
☐ At risk for mental illness
☐ Diagnosed with diabetes
☐ Diagnosed with heart disease
☐ Diagnosed with high blood pressure
☐ Diagnosed with cancer
☐ Diagnosed with mental illness
☐ Other ___________________

27 If yes, what motivates you to record physical activity/exercise for the patients you indicated?
(CHECK ALL THAT APPLY)

☐ Importance to health
☐ A program that you refer patients to
☐ Quality Incentives
☐ Other __________

28 Which measures do you use to assess exercise levels of your patients?
(CHECK ALL THAT APPLY)
If none, skip to #33 (next page)

☐ Physical fitness level
☐ Activity level
☐ None

		1	2	3	4	5
**29**	If any, what percentage of patients do you assess? (CIRCLE ONE)	1-20%	21-40%	41-60%	61-80%	81-100%

30 If any, what measure(s) of fitness assessment do you use?
(CHECK ALL THAT APPLY)

☐ Resting heart rate (Step test)
☐ Body Composition
☐ Something else _____________
☐ Number of push-ups or sit-ups
☐ BMI

31 If yes, what measure(s) of activity assessment do you use? (CHECK ALL THAT APPLY)

☐ Steps/day
☐ Activity monitor/device
Which device?_______________
☐ Something else _______________
☐ Perceived levels of exertion
☐ Minutes of activity per day/week
☐ Specific activity (walking, cycling, etc.)

32 If yes, with which target group(s) of patients do you assess physical activity/exercise? (CHECK ALL THAT APPLY)

☐ All patients	☐ Diagnosed with diabetes
☐ At risk for diabetes	☐ Diagnosed with heart disease
☐ At risk for heart disease	☐ Diagnosed with high blood pressure
☐ At risk for high blood pressure	☐ Diagnosed with cancer
☐ At risk for cancer	☐ Diagnosed with mental illness
☐ At risk for mental illness	☐ Other ___________________

**33**	Do you refer your patients to other professionals for fitness assessment and/or prescription? (CIRCLE ONE) **If no, skip to #37**				Yes	No
**34**	If yes, what percentage of patients do you refer?	1<br>1-20%	2<br>21-40%	3<br>41-60%	4<br>61-80%	5<br>81-100%

35 If yes, with which target group(s) of patients do you refer? (CHECK ALL THAT APPLY)

☐ All patients	☐ Diagnosed with diabetes
☐ At risk for diabetes	☐ Diagnosed with heart disease
☐ At risk for heart disease	☐ Diagnosed with high blood pressure
☐ At risk for high blood pressure	☐ Diagnosed with cancer
☐ At risk for cancer	☐ Diagnosed with mental illness
☐ At risk for mental illness	☐ Other ___________________

36 If yes, to which types of fitness professionals do you refer, for the purpose of physical fitness assessment or prescription? (CHECK ALL THAT APPLY)

☐ Physiotherapist
☐ Chiropractors
☐ Exercise Physiologist
☐ Personal Trainer
☐ Kinesiologists
☐ Other(s)________

**37**	Do you refer your patients to specific locations to engage in exercise? **If no, skip to #41**				Yes	No
**38**	If yes, what percentage of patients do you refer to a location?	1<br>1-20%	2<br>21-40%	3<br>41-60%	4<br>60-80%	5<br>81-100%

39 If yes, with what target group(s) of patients do you refer to specific locations? (CHECK ALL THAT APPLY)

☐ All patients
☐ At risk for diabetes
☐ At risk for heart disease
☐ At risk for high blood pressure
☐ At risk for cancer
☐ At risk for mental illness
☐ Diagnosed with diabetes
☐ Diagnosed with heart disease
☐ Diagnosed with high blood pressure
☐ Diagnosed with cancer
☐ Diagnosed with mental illness
☐ Other __________________

40 If yes, to which types of locations do you refer, for the purpose of physical activity or exercise? (CHECK ALL THAT APPLY)

☐ Hospital-owned fitness facility
☐ Walking or cycling to work/school
☐ Community-based center
☐ Park or trails
☐ Patient home, neighborhood, or worksite (videos, walking)
☐ Other(s) __________________________________

41 What would increase your number of referrals OR your likelihood of referring patients to a hospital-owned fitness facility (HOFF) (RANK IN ORDER OF IMPORTANCE, 1-6, 1 most important, 6 least important)

__ Informational materials
__ Increased insurance coverage
__ Increased training of HOFF staff
__ Your increased planning /approving
__ Incentive schemes
__ Something else_________________

42 If you DO NOT refer to hospital-owned fitness facilities (HOFF) (CHECK ALL THAT APPLY)

☐ Do not have a HOFF
☐ You are unaware of specific HOFF programs
☐ Patient safety concern
☐ Something else ___________________________

43 If you DO refer to hospital-owned fitness facilities, does your organization have a referral program?
(CHECK ONE)

☐ Yes
Name of program:_____________________________
☐ No

44 What would increase your number of referrals OR your likelihood of referring patients to parks/trails to exercise (CHECK ALL THAT APPLY)

☐ Park/trail informational materials (such as maps via handouts or websites)
☐ Increased medical supervision at park/trail
☐ Increased security (police)
☐ Increased park/trail quality
☐ Something else ____________

**45**	Exercise prescriptions are defined, like all prescriptions are, as having a type and dose, dosing frequency, a duration of treatment as a therapeutic goal, anticipated adverse effect, and written as a script.<br><br>Do you provide written physical activity/exercise prescriptions, as defined above, to your patients? (CHECK ONE)<br><br>☐ Yes ☐ No (**Skip to question #48)**					
**46**	If yes, to what percent of patients do you give written physical activity prescriptions?	1<br>1-<br>20%	2<br>21-<br>40%	3<br>41-<br>60%	4<br>61-<br>80%	5<br>81-<br>100%
**47**	If yes, with what target group(s) of patients do you give exercise prescriptions? (CHECK ALL THAT APPLY)<br>☐ All patients<br>☐ At risk for diabetes<br>☐ At risk for heart disease<br>☐ At risk for high blood pressure<br>☐ At risk for cancer<br>☐ At risk for mental illness<br>☐ Diagnosed with diabetes<br>☐ Diagnosed with heart disease<br>☐ Diagnosed with high blood pressure<br>☐ Diagnosed with cancer<br>☐ Diagnosed with mental illness<br>☐ Other ___________________					
	**<u>Barriers to Exercise Prescription</u>**<br><br>How important do you perceive the following barriers to be that might prevent you from prescribing exercise (more) regularly?	Circle a number for each item below.<br><br>**Disagree Strongly**	**Disagree Slightly**	**Neutral**	**Agree Slightly**	**Agree Strongly**
**48**	I do not have enough patient visit time	1	2	3	4	5
**49**	Patients' lack of time	1	2	3	4	5
**50**	Patients' lack of motivation/interest to change	1	2	3	4	5
**51**	Lack of standard guidelines on exercise counselling and prescription	1	2	3	4	5
**52**	Patients prefer pharmaceutical interventions	1	2	3	4	5
**53**	Lack of process in place for me to follow	1	2	3	4	5
**54**	Lack of evidence for health benefits of physical activity	1	2	3	4	5

**55**	I do not have enough reimbursement/ financial incentive	1	2	3	4	5
**56**	Lack of exercise education in medical school and residency	1	2	3	4	5
**57**	Lack of continuing education in physical activity	1	2	3	4	5
**58**	I need more personal knowledge	1	2	3	4	5
**59**	Other lifestyle changes are more important	1	2	3	4	5
**60**	Lack of safe locations to refer patients	1	2	3	4	5
**61**	Lack of affordable locations to refer patients	1	2	3	4	5
**62**	Lack of referral proximate to the patents in treatment	1	2	3	4	5
**63**	Lack of patients' cultural acceptance of physical activity/exercise prescriptions	1	2	3	4	5
**64**	Weight loss as the main focus of physical activity/exercise physical activity/exercise discourages patients	1	2	3	4	5
**65**	List other barriers you deem important:____					
	**<u>Continuing education needs assessment</u>**					
**66**	Have you received formal training about how to prescribe exercise? (CIRCE ONE) If yes, please include from which organization or institution you received the training.	Yes, ____	No			

67 What organization(s) do you earn your continuing education through? (CHECK ALL that apply)

☐ AAFP ☐ ACP

☐ AANP ☐ Other ______________

68 Topics of interest for more training in relation to exercise that would be useful (CHECK ALL that are of interest)

☐ Exercise prescription relative to specific medical conditions
☐ Exercise prescription relative to age
☐ Exercise prescription relative to specific medications
☐ Locations to refer patients
☐ Other___________________________________

69 Training format (Rank in order of preference, 1 most preferred, 5 least preferred.)

__ Face-to-face seminar/conference
__ Video/DVD
__ Printed self-study guide
__ Online/internet training
__ Something else_________________

70 Materials to share/administer to patients (Rank in order of preference, 1 most preferred, 5 least preferred.)

__ Poster-based guidelines for wall
__ Prescription pad for exercise
__ Video
__ Online/internet
__ Phone/tablet application
__ Something else__________

These questions are about your physical activity.

<u>International Physical Activity Questionnaire – Short Form</u>

We are interested in finding out about the kinds of physical activities that people do as part of their everyday lives. The questions will ask you about the time you spent being physically active in the **<u>last 7 days</u>**. Please answer each question even if you do not consider yourself to be an active person. Please think about the activities you do at work, as part of your house and yard work, to get from place to place, and in your spare time for recreation, exercise or sport.

Think about all the **vigorous** activities that you did in the **last 7 days**. **Vigorous** physical activities refer to activities that take hard physical effort and make you breathe much harder than normal. Think *only* about those physical activities that you did for at least 10 minutes at a time

71 During the **last 7 days**, on how many days did you do **vigorous** physical activities like heavy lifting, digging, aerobics, or fast bicycling?

____ days per week ☐ No vigorous physical activities.
If none, Skip **to question 73**

72 How much time did you usually spend doing vigorous physical activities on one of those days?

_____ hours per day _____ minutes per day ☐ Don't know/Not sure

73 Think about all the moderate activities that you did in the last 7 days. Moderate activities refer to activities that take moderate physical effort and make you breathe somewhat harder than normal. Think only about those physical activities that you did for at least 10 minutes at a time.

During the last 7 days, on how many days did you do moderate physical activities like carrying light loads, bicycling at a regular pace, or doubles tennis? Do not include walking.

_____ days per week ☐ No moderate physical activities.
If none, Skip to question 75

74 How much time did you usually spend doing moderate physical activities on one of those days?

_____ hours per day _____ minutes per day ☐ Don't know/Not sure

75 Think about the time you spent walking in the last 7 days. This includes at work and at home, walking to travel from place to place, and any other walking that you have done solely for recreation, sport, exercise, or leisure.

During the last 7 days, on how many days did you walk for at least 10 minutes at a time?

_____ days per week ☐ No walking. **If none, Skip to question 77**

76 How much time did you usually spend walking on one of those days?

_____ hours per day _____ minutes per day ☐ Don't know/Not sure

77 The last question is about the time you spent sitting on weekdays during the last 7 days. Include time spent at work, at home, while doing course work and during leisure time. This may include time spent sitting at a desk, visiting friends, reading, or sitting or lying down to watch television.

During the last 7 days, how much time did you spend sitting on a week day?

_____ hours per day _____ minutes per day ☐ Don't know/Not sure

Demographic information:

78 With which gender do you identify? (CHECK ONE)

☐ Male ☐ Female

79 What is your ethnic/racial background? (CHECK ALL THAT APPLY)

☐ White ☐ Black or African American
☐ Native American or American Indian ☐ Asian / Pacific Islander
☐ Choose not to answer ☐ Hispanic or Latino

80 What is your age? ____________

81 What is your specialty/role? (CIRCLE ONE)

☐ Family Medicine ☐ Internal Medicine ☐ Nurse Practitioner
☐ Other _______

82 Are you currently a final year resident? (CIRCE ONE) Yes No

83 How many years have you been practicing medicine _____________ years

84 Where did you receive your exercise/physical activity medical training?

☐ CME ☐ Medical School ☐ It was not included
☐ Other_______

85 What is your practice location? (CHECK ALL THAT APPLY)

☐ Rural ☐ Urban ☐ Suburban

86 What is the structure of your practice? (CHECK ONE)

☐ Solo practice ☐ Group practice ☐ Hospitalist
☐ Other ______

87 Are you part of an Accountable Care Organization (ACO), wellness, or population health quality initiative? (CHECK ONE) If yes, please include the initiative.

☐ Yes ____ ☐ No

88 What hospital(s) do you work and/or have admitting privileges?
____
